# The α-melanocyte stimulating hormone/peroxisome proliferator activated receptor-γ pathway down-regulates proliferation in melanoma cell lines

**DOI:** 10.1186/s13046-017-0611-4

**Published:** 2017-10-11

**Authors:** Enrica Flori, Eleonora Rosati, Giorgia Cardinali, Daniela Kovacs, Barbara Bellei, Mauro Picardo, Vittoria Maresca

**Affiliations:** Laboratory of Cutaneous Physiopathology and Integrated Center of Metabolomics Research, San Gallicano Dermatologic Institute (IRCCS), Via Elio Chianesi 53, 00144 Rome, Italy

**Keywords:** αMSH, MC1R, PPARγ, cAMP/PKA pathway, Phosphatidylinositol signaling pathway, Proliferation, Cell cycle, melanocytes, Melanoma

## Abstract

**Background:**

The α-Melanocyte Stimulating Hormone (αMSH)/Melanocortin-1 receptor (MC1R) interaction promotes melanogenesis through the cAMP/PKA pathway. The direct induction of this pathway by Forskolin (FSK) is also known to enhance melanocyte proliferation. αMSH acts as a mitogenic agent in melanocytes and its effect on proliferation of melanoma cells is less known. We previously identified the αMSH/Peroxisome Proliferator Activated Receptor (PPARγ) pathway as a new pathway on the B16-F10 mouse melanoma cell line. αMSH induced the translocation of PPARγ into the nucleus as an active transcription factor. This effect was independent of the cAMP/PKA pathway and was mediated by the activation of the PI(4,5)P2/PLC pathway, a pathway which we have described to be triggered by the αMSH-dependent MC1R stimulation. Moreover, in the same study, preliminary experiments showed that mouse melanoma cells responded to αMSH by reducing proliferation and that PPARγ was involved in this effect. Due to its key role in the control of cell proliferation, PPARγ agonists are used in therapeutic models for different forms of cancer, including melanoma. The purpose of this study was: (a) to confirm the different proliferative behavior in response to αMSH in healthy and in melanoma condition; (b) to verify whether the cAMP/PKA pathway and the PLC/PPARγ pathway could exert an antagonistic function in the control of proliferation; (c) to deepen the knowledge of the molecular basis responsible for the down-proliferative response of melanoma cells after exposure to αMSH.

**Methods:**

We employed B16-F10 cell line, a human melanoma cell line (Mel 13) and two primary cultures of human melanocytes (NHM 1 and NHM 2, respectively), all expressing a wild type MC1R and responding to the αMSH in terms of pigmentation. We evaluated cell proliferation through: a) cell counting, b) cell cycle analysis c) protein expression of proliferation modulators (p27, p21, cyclin D1 and cyclin E).

**Results:**

The αMSH acted as a mitogenic agent in primary cultures of human melanocytes, whereas it determined a slow down of proliferation in melanoma cell lines. FSK, as an inducer of the cAMP/PKA pathway, reproduced the αMSH mediated effect on proliferation in NHMs but it did not mimic the αMSH effect on proliferation in B16-F10 and Mel 13 melanoma cell lines. Meanwhile, 3 M3-FBS (3 M3), as an inducer of PI(4,5)P2/PLC pathway, reproduced the αMSH proliferative effect. Further experiments, treating melanoma cell lines with αMSH in the presence/absence of GW9662, as an inhibitor of PPARγ, confirmed the key role of this transcription factor in decreasing cell proliferation in response to the hormone exposure.

**Conclusions:**

In both melanoma cell lines, αMSH determined the reduction of proliferation through the PI(4,5)P2/PLC pathway, employing PPARγ as an effector element. These evidence could offer perspectives for new therapeutic approaches for melanoma.

**Electronic supplementary material:**

The online version of this article (10.1186/s13046-017-0611-4) contains supplementary material, which is available to authorized users.

## Background

The Melanocortin-1 receptor (MC1R), expressed on the surface of melanocytes and melanoma cells, and its ligand, the α-Melanocyte Stimulating Hormone (αMSH), represent a key interaction in the control of melanogenesis [1–4]. MC1R activation stimulates cAMP synthesis via G-protein, which in turn mediates the phosphorylation of the cAMP responsive element-binding protein (CREB) transcription factor. In turn, CREB participates in the activation of Micropthalmia Transcription Factor (MITF), the crucial transcription factor for melanocyte differentiation and melanogenesis [5, 6].

We previously identified a new pathway between αMSH and Peroxisome Proliferator Activated Receptor (PPARγ) on B16-F10 mouse melanoma cell line [7]. In the same work we demonstrated that αMSH induced the translocation of PPARγ into the nucleus. This effect was not reproduced by Forskolin (FSK) (a direct activator of the cAMP/PKA pathway by [8]) and was dependent on the activation of the PI(4,5)P2/PLC pathway. Moreover, a preliminary experiment showed that the B10-F10 cell line responded to αMSH by reducing proliferation and that PPARγ was directly involved in this effect. PPARγ is a transcription factor capable of promoting pleyotropic effects, including the control of proliferation [9, 10]. In fact, many studies in cancer therapy employ pharmacological agonists of PPARγ with the aim of promoting a slow down of the cell cycle [11, 12].

Only sporadic and very old studies have dealt with the capacity of activated MC1R to modulate proliferation in melanoma cells and melanocytes: transformed cells respond to αMSH by proliferating or down-regulating proliferation, according to the degree of pigmentation and the progression of the pathology [13, 14]; primary cultures of human melanocytes respond to αMSH with a hyper-proliferative behaviour [1, 3, 15–17] and some authors have defined αMSH as a mitogenic agent for melanocytes [1, 3]. The mechanism promoting hyper-proliferation in melanocytes could be recognized in the cAMP/PKA pathway itself (the canonical pathway triggered by the activation of MC1R). In fact, cAMP elevating agents, such as FSK, enhance the proliferation and melanogenesis of cultured human melanocytes [18–20].

Here we worked on B16-F10 murine melanoma, on Mel 13 human melanoma cell line and on primary human melanocytes NHM 1 and NHM 2. All human cell lines were set up in our laboratory starting from biopsy explants. All cell lines expressed a wild type MC1R and responded to αMSH in terms of pigmentation (see Methods). We aimed to confirm the different proliferative effects, in response to αMSH, in melanoma and in healthy condition. Moreover we proposed to verify whether the cAMP/PKA pathway and the PLC/PPARγ pathway could exert an antagonistic function in the control of proliferation. Furthermore, we aimed to deepen the knowledge of the mechanism responsible for the down-proliferative response of melanoma cells after exposure to αMSH, by analyzing cell proliferation and protein levels of p27, p21, cyclin D1 and E, as typical proliferation modulators. Cyclin D1 and E and their associated cyclin-dependent kinases (CDKs: CDK4 and CDK6 for cyclin D1; CDK2 for cyclin E) are central mediators in transition from G1 and S phase [21, 22]. On the contrary, p21 and p27 represents two G1-check point CDK inhibitors [23–25].

Our previous study mainly underlined the existence of the αMSH/PPARγ pathway in association with the canonical cAMP/PKA, but only started to deal with its effects on proliferation. Moreover, it essentially employed the B16-F10 murine melanoma cell line [7]. The present study extended the analysis of the αMSH/PPARγ pathway to human cell lines. The novelty of this work was to focus on the PLC/PPARγ and cAMP/PKA pathways on cell proliferation. In summary, on cell lines expressing a wild type MC1R and responding to αMSH in terms of pigmentation, this study confirmed the hyper-proliferative effect of αMSH in primary cultures of melanocytes and the opposite effect in melanoma cell lines. Moreover, it associated an antagonistic function to the cAMP/PKA and the PLC/PPARγ pathways in the control of proliferation. Finally, it highlighted that the reduction of proliferation, observed in melanoma cell lines exposed to αMSH, showed characteristics of cell cycle withdrawal. A more in-depth analysis of these mechanisms could provide useful information for the development of innovative therapeutic strategies for melanoma.

## Methods

### Cell lines and treatments

B16-F10 cell line is a classical melanoma cell line widely employed in pigmentation studies, because it expresses a wild type MC1R, with an intact transduction machinery, which is activated in response to receptor stimulation [26]. B16-F10 mouse melanoma cells were cultured in Dulbecco’s modified Eagle’s medium (DMEM) supplemented with heat-inactivated 7% fetal bovine serum (FBS) and antibiotics (all products purchased by EuroClone, Milan, Italy). The human Mel13-SM melanoma cell line (Mel 13) was isolated in our laboratory as previously reported [27], exclusively from excess parts of the biopsy collected for histological examination, without compromising the standard diagnostic procedure. Mel 13 derived from a skin metastasis removed from the leg of a 52-year-old male (pT3bN0M1, stage IV). Mel 13 cell line was grown in OptiMEM (Invitrogen Life Technologies Italia, Monza, Italy) medium containing 10% FBS and antibiotics. All the experiments were performed at low cell culture passage. Two primary human melanocyte cultures, NHM 1 and NHM 2 respectively (NHMs), were set up in our laboratory from neonatal foreskin in accordance with a previously described procedure [28]. NHMs were selectively grown in the defined medium M254 (Invitrogen Life Technologies Italia, Monza, Italy) and Human melanocyte growth supplements (HMGS) (Invitrogen Life Technologies Italia, Monza, Italy). NHMs were sub-cultured once a week and experiments carried out on cells between passages 2 to 6.

B16-F10 cell cultures and human melanoma cells were plated and 24 h later were stimulated with chemicals in fresh medium. Primary human melanocytes were plated and 24 h later were stimulated with chemicals in a fresh medium deprived of bovine pituitary extract (BPE) and phorbol 12-myristate 13-acetate (PMA). The following doses of chemicals were employed: 10^−7^ M αMSH (Sigma-Aldrich Srl, Milan, Italy); 3 μM GW9662 (Sigma-Aldrich Srl, Milan, Italy) a potent and irreversible antagonist of PPARγ [29]; 15 μM 3 M3-FBS (3 M3) (Merck KGaA, Darmstadt, Germany), a PLC inducer [30]; 1 μM Forskolin (FSK) (Sigma- Sigma-Aldrich Srl, Milan, Italy), a cAMP-stimulating agent [8].

### Ethical statement

Institute Research Ethics Committee (IFO) approval was obtained to collect samples of human material for research. The Declaration of Helsinki Principles was followed, and patients gave written informed consent. For biopsies obtained from minors written consent was given by the parents or legal guardians.

### RNA extraction and quantitative real-time RT-PCR

Total RNA was isolated using the Aurum™ Total RNA Mini kit (Bio-Rad Laboratories Srl, Milan, Italy). Following DNAse I treatment, cDNA was synthesized using oligo-dT primers and using ImProm-II™ Reverse Transcriptase (Promega Corporation, Madison, WI) according to the manufacturer’s instructions. Quantitative real time RT-PCR was performed in a total volume of 15 μl with SYBR Green PCR Master Mix (Bio-Rad Laboratories Srl) and 200 nM concentration of each primer. The primer sequences were as follows: hMC1R sense: 5′-CCTGAAGACCTCACTAGG -3′ and antisense: 5′-CATCTTGTAGAGCCTGAG-3′; mMC1R sense: 5′-CAAGGAGGTGCTGCTGTG-3 and antisense: 5′-TAGACAAATGGAGATCAGGAAGG-3′, β-actin sense: 5′-GACAGGATGCAGAAGGAGATTACT -3′ and antisense 5′-TGATCCACATCTGCTGGAAGGT-3′. Reactions were carried out in triplicate using the Real-Time Detection System iQ5 (Bio-Rad Laboratories Srl) supplied with iCycler IQ5 optical system software version 2.0 (Bio-Rad Laboratories Srl). Melt curve analysis was performed to confirm the specificity of the amplified products. β-actin was used as an endogenous control.

### Immunofluorescence analysis

Cells grown on coverslips previously coated with 2% gelatin on 24-well plates were treated with 3 M3 (15 μM) for 1, 3 and 6 h. For immunolabelling with anti-PPARγ rabbit antibody (1:50 in PBS) (Cell Signalling Tecnology, New England Biolabs, UK) cells were fixed in methanol for 4 min. at −20 °C. The primary antibody was visualized by using anti-rabbit IgG- Alexa Fluor 555 conjugated antibody (1:800 in PBS) (Cell Signaling, Tecnology, New England Biolabs, UK). Nuclei were visualized with DAPI (Sigma-Aldrich Srl). Fluorescence signals were analyzed by scanning cells in a series of sequential sections with an ApoTome System (Zeiss, Oberkochen, Germany); image analysis was performed by the Axiovision software (Zeiss) and 3D reconstruction of a selection of three central optical sections was shown in each image. Quantitative analysis of the extent of PPARγ/DAPI colocalization was determined using Axiovision colocalization module (Zeiss) analyzing 100 cells for each condition randomly taken from 10 different microscopic fields. Results are expressed as fold increase of colocalization signals with respect to the values obtained in untreated cells and are reported as mean values ± SD (%). Three distinct experiments were analyzed. For immunolabelling with anti-MC1R (N-19) goat antibody (1:50 in PBS) (Santa Cruz Biotechnology, INC) cells were fixed in PFA 4% for 15 min at room temperature. The primary antibody was visualized using anti-goat IgG-Alexa Fluor 488 conjugated antibody (1:800 in PBS) (ThermoFisher Scientific). Nuclei were visualized with DAPI (Sigma-Aldrich Srl).

### Western blot analysis

Cells were lysed in denaturing conditions supplemented with a protease inhibitor cocktail (Roche, Mannheim, Germany). The protein concentration of extracts was estimated with Bradford reagent (Bio-Rad Laboratories Srl, Milan, Italy). Equal amounts of proteins were then separated on acrylammide SDS-PAGE, transferred onto nitrocellulose (Amersham Biosciences, Milan, Italy) and then treated overnight at 4 °C with: anti-tyrosinase antibody, anti-cyclin E antibody (1:1000; Santa Cruz Biotechnology Inc., Santa Cruz, CA, USA); anti-p27 antibody, anti-p21 antibody, anti-cyclin D1 antibody (all 1:1000; Cell Signalling Technology, New England Biolabs, UK). HRP-conjugated goat anti-mouse (1:3000; Cell Signalling Technology) or anti-rabbit IgG (1:8000; Cell Signalling Technology) were used as secondary antibodies. Antibody complexes were visualized using the an enhanced chemiluminescence reagent (ECL) (Amersham Biosciences, Milan, Italy). A subsequent hybridization with anti-GAPDH or anti-HSP70 (both 1:5000; Santa Cruz Biotechnology Inc., Santa Cruz, CA, USA) was used to estimate the equal protein loading. Densitometric analysis was performed using a GS-800 Calibrated Image Densitometer (Bio-Rad Laboratories Srl, Milan, Italy) or with UVITEC Imaging System (Cambridge, UK). Results refer to three independent experiments.

### Cell number analysis

Cells were plated in a 12 well plate at a density of 1 X 10^4^ cells/well and were grown overnight. Cells were treated with 10^−7^ M αMSH, 3 μM GW9662, 15 μM 3 M3 and 1 μM FSK at different time points, in accordance with the experimental design. Cell number was determined by cell counting using a phase contrast microscope. None of the employed doses of chemicals determined positivity to the Trypan blue exclusion assay test. The results presented are the average of three experiments in triplicate.

### Flow cytometric analysis of cell cycle

Cells were harvested by treatment with 0.25% trypsin, fixed with ice-cold 70% ethanol solution, and stained in PBS containing propidium iodide (100 μg/ml) and RNase A (90 μg/ml) overnight at 4 °C. The DNA content of the cells was measured by FACScalibur flow cytometer (Becton Dickinson, Franklin Lakes, NJ, USA). A total number of 10^4^ cells for each sample were acquired. Flow cytometric histograms were analyzed by defining borders between pre-G_1_, G_1_, S and G_2_ + M phase populations. Cell Quest Software was used to analyze data.

### Transfection and luciferase assay

Cells were transfected with pGL3-(Jwt) 3TKLuc reporter construct [31] using specific Amaxa® Nucleofector kits (Lonza, Basel, Switzerland) according to the manufacturer’s instructions. 24 h after treatment with 10^−7^ M αMSH or 15 μM 3 M3, cells were harvested in 100 μl lysis buffer and 20 μl of the extract were assayed for luciferase activity using Promega’s Dual Luciferase (Promega Corporation, Madison, WI, USA) according to the manufacturer’s protocol. The renilla luciferase plasmid was also transfected as an internal control for monitoring transfection efficiency and for normalizing the firefly luciferase activity. The luciferase activity was expressed as fold of the activity obtained in cells treated with the different molecules divided by luciferase activity from non-stimulated cells. The pGL3-(Jwt)3TKLuc reporter construct is described elsewhere [31]. The results represent the average of three independent experiments performed in triplicate.

### Statistical analyses

Data are presented as means ± SD. The Student’s t-test was used to analyze differences. Values of *p* < 0.05 were considered significant.

## Results

### αMSH reduced melanoma cell proliferation while it exerted a mitogenic effect in primary human melanocytes

**Fig. 1:**
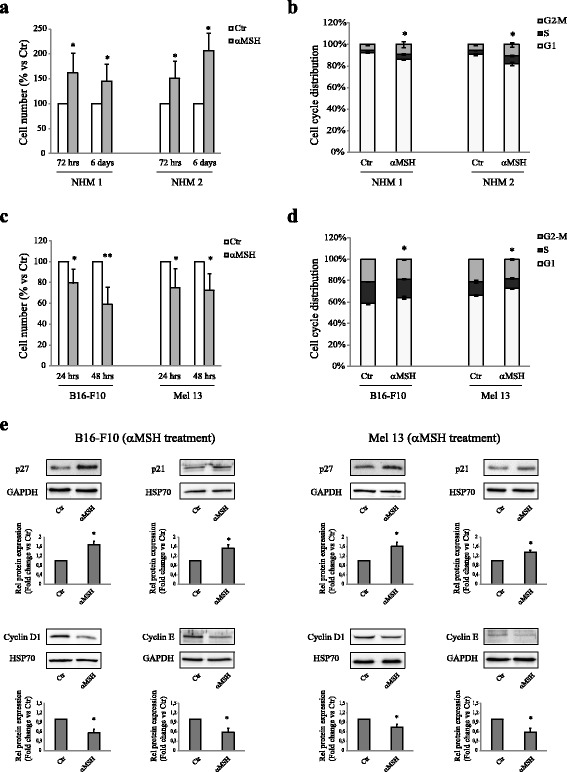
αMSH acts as a mitogenic agent in NHMs, whereas it determined a slow down of proliferation in B16-F10 and Mel 13 melanoma cell lines. **a** Analysis of cell number performed on NHM 1 and NHM 2, after treatment with 10^−7^ M αMSH for 72 h and 6 days. Cell number was expressed as a percent variation in comparison to the value of untreated control cells. Data are mean values ± SD of three independent experiments performed in triplicate. *p < 0.01 (vs untreated cells). **b** Cell-cycle distribution was evaluated by flow cytometric analysis on NHM 1 and NHM 2, after treatment for 48 h with 10^−7^ M αMSH. The bar graph shows the distribution of cells among the different phases of the cell cycle. Data are mean values ± SD of three independent experiments performed in duplicate. *p < 0.01 (vs untreated cells). **c** Analysis of cell number performed on B16-F10 and Mel 13, after treatment with 10^−7^ M αMSH for 24 and 48 h. Cell number was expressed as a percent variation in comparison to the value of untreated control cells. Data are mean values ± SD of three independent experiments performed in triplicate. *p < 0.01 and **p < 0.001 (vs untreated cells). **d** Cell-cycle distribution evaluated by flow cytometric analysis on B16-F10 and Mel 13, after treatment for 24 h with 10^−7^ M αMSH. The bar graph shows the distribution of cells among the different phases of the cell cycle. Data are mean values ± SD of three independent experiments performed in duplicate. *p < 0.01 (vs untreated cells). **e** Western blot analysis of p27 (4 h), p21 (48 h), cyclin D1 and cyclin E (6 h) protein expression on cell lysate of B16-F10 and Mel 13, treated with 10^−7^ M αMSH. GAPDH or HSP70 were used as an equal loading control. Results refer to three independent experiments. Representative blots are shown. Densitometric scanning of band intensities was performed to quantify the change of protein expression (control value taken as one fold in each case). ^*^ *p* < 0,01 (vs untreated cells)

We investigated the capacity of αMSH to modulate proliferation in primary human melanocytes NHM 1 and NHM 2, in B16-F10 murine melanoma cell line and in the Mel 13 human melanoma cell line. All these cell lines were characterized by the genetic nature of MC1R, expressing a wild type MC1R and responding to αMSH in terms of pigmentation (see Additional file 1 and Additional file 2: Figure S1). The duplication time of primary cultures of human melanocytes is significantly longer with respect to that of B16-F10 and Mel 13 melanoma cell line (30/40 h vs 18/24 h). Thus, we planned to perform evaluations at suitable times to highlight variations in treated samples in comparison with untreated ones. The exposure to 10^−7^ M αMSH for 3 and 6 days, significantly increased the cell number in both NHMs (Fig. 1a). Accordingly, 48 h hrs of treatment with the hormone significantly decreased the proportion of cells distributed in the G0/G1 phase of the cell cycle (Fig. 1b). Instead, αMSH was unable to act as a mitogenic agent either in B16-F10 or Mel 13 cell lines, and it even reduced the cell number. This effect was already significant after 24 h of treatment (Fig. 1c). In compliance with these results, 24 h of treatment with αMSH determined a significant increase in the proportion of cells which were distributed in the G0/G1 phase of the cell cycle (Fig. 1d). In order to corroborate the αMSH effect on proliferation slow down, we evaluated the expression levels of G1 phase related proteins (p27, p21, cyclin D1 and E), in response to the hormone exposure. In preliminary experiments, expression levels of p27, p21, cyclin D1 and E, after exposure to αMSH, or alternative stimuli (see below), were followed at 4, 6, 12, 24, 36 and 48 h (data not shown). Here, and in the subsequent experiments (also see following paragraphs), we reported data associated with times of maximal proteins modulation. In both melanoma cell lines, the expression level of the cyclin-dependent kinase inhibitor p27 already resulted significantly up-regulated after 4 h of treatment with αMSH. The same treatment determined a significant induction of the expression level of the cyclin-dependent kinase inhibitor p21 after 48 h. Consistently, αMSH exposure significantly down-regulated the expression of both cyclin D1 and E after 6 h (Fig. 1e). Thus, these results indicate that αMSH exerted a mitogenic effect in primary human melanocytes while it reduced melanoma cell proliferation.

### Analysis of cAMP/PKA pathway in the control of NHMs and melanoma cell line proliferation

**Fig. 2:**
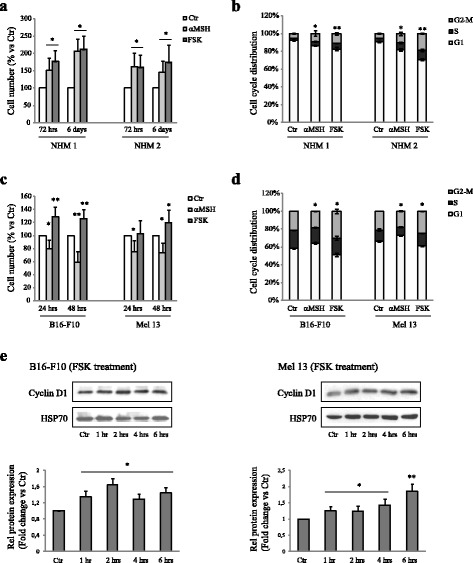
The direct induction of the cAMP/PKA pathway by Forskolin mimics the αMSH mediated effect on proliferation in NHMs but it does not reproduce the αMSH mediated effect on proliferation in B16-F10 and Mel 13 melanoma cell lines. **a** Analysis of cell number performed on NHM 1 and NHM 2, after treatment with 10^−7^ M αMSH or 1 μM FSK for 72 h and 6 days. Cell number was expressed as a percent variation in comparison to the value of untreated control cells. Data are mean values ± SD of three independent experiments performed in triplicate. *p < 0.01 (vs untreated cells). **b** Cell-cycle distribution evaluated by flow cytometric analysis on NHM 1 and NHM 2, after treatment for 48 h with 10^−7^ M αMSH or 1 μM FSK . The bar graph shows the distribution of cells among the different phases of the cell cycle. Data are mean values ± SD of three independent experiments performed in duplicate. *p < 0.01 and **p < 0.001 (vs untreated cells). **c** Analysis of cell number performed on B16-F10 and Mel 13, after treatment with 10^−7^ M αMSH or 1 μM FSK for 24 and 48 h. Cell number was expressed as a percent variation in comparison with the value of untreated control cells. Data are mean values ± SD of three independent experiments performed in triplicate. *p < 0.01 and **p < 0.001 (vs untreated cells). **d** Cell-cycle distribution evaluated by flow cytometric analysis on B16-F10 and Mel 13, after treatment for 24 h with 10^−7^ M αMSH or 1 μM FSK. The bar graph shows the distribution of cells among the different phases of the cell cycle. Data are mean values ± SD of three independent experiments performed in duplicate. *p < 0.01 (vs untreated cells). **e** Western blot analysis of Cyclin D1 protein expression on cell lysate of B16-F10 and Mel 13, treated with FSK for 1, 2, 4 and 6 h. HSP70 was used as an equal loading control. Results refer to three independent experiments. Representative blots are shown. Densitometric scanning of band intensities was performed to quantify the change of protein expression (control value taken as one fold in each case). ^*^ *p* < 0,01 and **p < 0.001 (vs untreated cells)

The cAMP/PKA pathway is the pathway through which the MC1R is universally known to promote melanin synthesis [2, 5]. FSK, as a cAMP elevating agent, mimics the effects of αMSH on melanogenesis [8]. Furthermore, FSK is employed in defined cell culture media to promote the proliferation of melanocytes [18–20]. Here, we verified whether FSK was able to mimic the effects induced by αMSH on proliferation in NHMs. In both NHMs, the treatment for 3 and 6 days with 1 μM FSK promoted a significant increase in cell number, similar to that produced by αMSH in the same experimental conditions (Fig. 2a). In compliance with these results, the exposure to FSK promoted a significant decrease in the proportion of cells distributed in the G0/G1 phase of the cell cycle (Fig. 2b). Thus, these results indicate that the αMSH-dependent mechanism was able to promote hyper-proliferation in NHMs can be recognized in the cAMP/PKA pathway.

In our previous study on B16-F10, in a preliminary experiment of cell counting, we demonstrated that αMSH induce a slow down of proliferation. This effect was mediated by the αMSH/PPARγ pathway, a pathway independent of the cAMP/PKA canonical pathway [7]. Here, we proposed to exclude the involvement of the cAMP/PKA pathway in mediating the proliferation slow down induced by αMSH in B16-F10 and Mel 13 melanoma cell lines. Both in B16-F10 and Mel 13, the treatment for 24 an 48 h with 1 μM FSK caused an increase in cell number, producing an opposite effect in comparison to that promoted by αMSH (Fig. 2c). The analysis of cell cycle phase distribution, after 24 h of treatment with FSK, corroborated these results (Fig. 2d). Moreover, a Western blot analysis of cyclin D1 showed a rapid induction of this key regulator of cell cycle progression (Fig. 2e). These results, confirmed our initial hypothesis. In fact, they excluded the involvement of the cAMP/PKA pathway in mediating the αMSH-mediated slow down of proliferation in B16-F10 and Mel 13 melanoma cell lines.

### The direct induction of the PI(4,5)P2/PLC pathway by 3 M3 mimics the αMSH mediated effect on proliferation in B16-F10 and Mel 13 melanoma cell lines

**Fig. 3:**
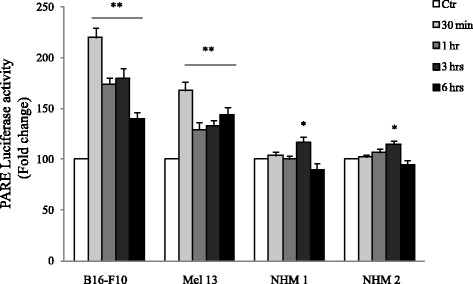
Analysis of PPARγ transcriptional activity in response to αMSH exposure in B16-F10, Mel 13 and NHMs. Cells were transfected with pGL3-(Jwt)3TKLuc reporter construct. After 24 h of transfection, cells were treated with 10^−7^ M αMSH. The measurement of luciferase activity was carried out 30 min, 1, 3 and 6 h after treatment. The variability of transfection was normalized with renilla luciferase activity. The results were expressed as fold change with respect to untreated cells. Data are mean values ± SD of three independent experiments performed in triplicate, *p < 0.01 and **p < 0.001 (vs untreated cells)

In an attempt to find an alternative pathway which could justify the proliferation slow down induced by αMSH in B16-F10 and Mel 13 melanoma cell lines, our attention focused on the PI(4,5)P2/PLC pathway [7]. PPARγ is a tumour suppressor gene [9, 10]. Here we show that the treatment with αMSH promoted a significant induction of PPARγ transcriptional activity after 24 h of exposure, in B16-F10, Mel 13 and NHMs. Moreover, this effect was consistently higher in melanoma cell lines compared to NHMs (Fig. 3). The αMSH/PPARγ connection is mediated by the PI(4,5)P2/PLC pathway. In turn, this pathway can be directly activated by 3 M3 [30]. Thus, we tried to understand whether the treatment with 3 M3 was able to mimic the down-proliferation induced by αMSH in B16-F10 and Mel 13 melanoma cells.

**Fig. 4:**
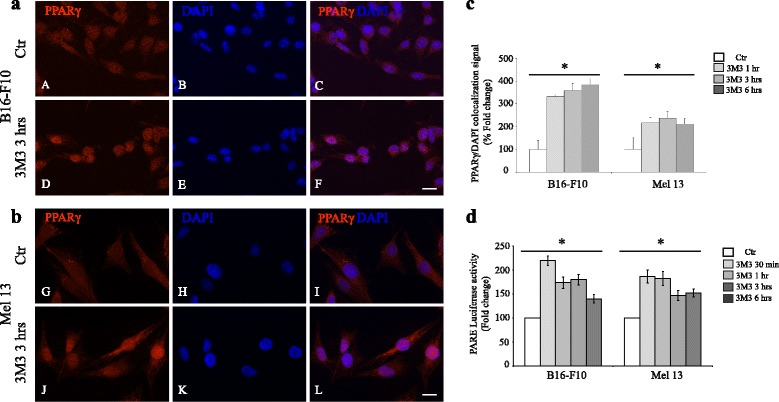
Analysis of PPARγ translocation into the nucleus and activity in response to 3 M3 exposure. (**a**, **b**, **c**) Immunofluorescence analysis of PPARγ localization in untreated cells (*A*-*C*, *G*-*I*) and in cells treated with 15 μM 3 M3 for 3 h (*D*-*F*, *J*-*L*). Immunolabeling with anti-PPARγ antibody and nuclear staining with DAPI. Scale bar: 20 μM. (**c**) Quantitative analysis of the PPARγ/DAPI colocalization signal in the nucleus. Results are express as fold increase of colocalization signal with respect to the values obtained in untreated cells and are reported as mean value ± SD (%) (*p < 0.01). (**d**) Transcriptional activity of PPARγ (fold change) by luciferase activity assay in B16-F10 and Mel 13. Cells were transfected with pGL3-(Jwt)3TKLuc reporter construct. After 24 h of transfection, cells were treated with 15 μM 3 M3. The measurement of luciferase activity was carried out 24 h after treatment. The variability of transfection was normalized with *β*-Gal activity. The results were expressed as fold change with respect to untreated cells. Data are mean values ± SD of three independent experiments performed in triplicate. *p < 0.01 (vs untreated cells)

Preliminary checks: first, we verified the capacity of 3 M3 to promote: (a) calcium fluxes (Additional file 3: Figure S2), (b) PPARγ translocation into the nucleus (Fig. 4a–c) and (c) PPARγ transcriptional activity by luciferase assay (Fig. 4d). (a) Concerning the analysis of calcium fluxes, the cells previously loaded with fluo-3-AM, responded to 3 M3 treatment with an increased intra-cytoplasmic calcium, which was significant at all time points evaluated. This increase consisted in an initial response, which reached a peak after 2 min, followed by a subsequent decline to plateau value after 10 min (Additional file 3: Figure S2). (b) We also evaluated the PPARγ nuclear translocation in response to 3 M3 at different time points: 1, 3 and 6 h, by immunofluorescence analysis. We observed a positive signal for PPARγ in both cytoplasm and nucleus, in untreated cells. We observed an increase of PPARγ nuclear staining in cells exposed to 3 M3, demonstrating a translocation of this transcription factor from the cytoplasm to the nucleus (Fig. 4a,b). This observation was also confirmed by the analysis of the extent of PPARγ/DAPI colocalization signals in the nucleus (Fig. 4c). (c) Luciferase assay, using the pGL3-(Jwt)3TKLuc reporter construct [31], consistently showed a significant induction of the PPARγ transcriptional activity after 24 h of 3 M3 treatment (Fig. 4d).

**Fig. 5:**
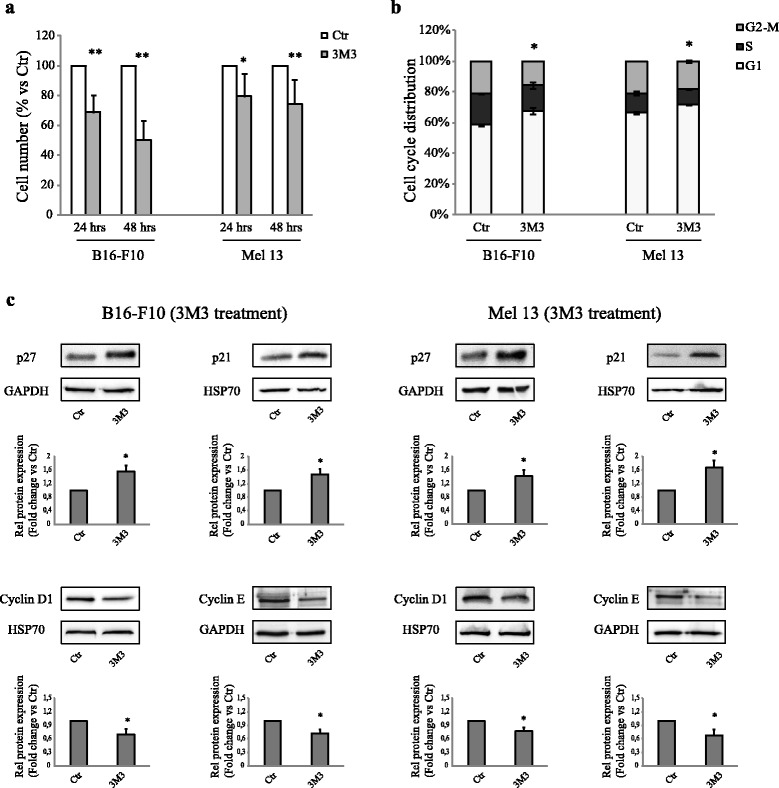
The direct induction of the pathway by 3 M3 mimics the αMSH mediated effect on proliferation in B16-F10 and Mel 13 melanoma cell lines. **a** Analysis of cell number performed on B16-F10 and Mel 13, after treatment with 15 μM 3 m3 for 24 and 48 h. Cell number was expressed as a percent variation in comparison with the value of untreated control cells. Data are mean values ± SD of three independent experiments performed in triplicate. *p < 0.01 and **p < 0.001 (vs untreated cells). **b** Cell-cycle distribution evaluated by flow cytometric analysis on B16-F10 and Mel 13, after treatment for 24 h with 15 μM 3 m3. The bar graph shows the distribution of cells among the different phases of the cell cycle. Data are mean values ± SD of three independent experiments performed in duplicate. *p < 0.01 (vs untreated cells). **c** Western blot analysis of p27 (4 h), p21 (48 h), cyclin D1 and cyclin E (6 h) protein expression on cell lysate of B16-F10 and Mel 13, treated with 15 μM 3 m3. GAPDH or HSP70 were used as an equal loading control. Results refer to three independent experiments. Representative blots are shown. Densitometric scanning of band intensities was performed to quantify the change of protein expression (control value taken as one fold in each case). ^*^ *p* < 0,01 (vs untreated cells)

Then, we tried to understand whether the treatment with 3 M3 was able to reproduce the effects of αMSH on cell number and proliferation in B16-F10 and Mel 13 cells. The exposure to 3 M3 for 24 and 48 h determined a significant decrease of cell number in both melanoma cell lines. This decrease was fully comparable to that promoted by αMSH (Fig. 5a). Furthermore, the exposure to 3 M3 for 24 h, promoted a significant increase in the proportion of cells distributed in the G0/G1 phase of cell cycle (Fig. 5b). The expression level of p27 was already significantly induced after 4 h of treatment with 3 M3, in both melanoma cell lines. The same treatment determined a significant up-regulation of p21 protein expression after 48 h. Consistently, 3 M3 exposure significantly down-regulated the levels of both cyclin D1 and cyclin E after 6 h (Fig. 5c). Thus, the direct induction of the pathway by 3 M3 is able to reproduce the αMSH mediated effect on proliferation in B16-F10 and Mel 13 melanoma cell lines.

### The PPARγ acts as an effector element in determining the reduction of cell number induced by the activation of PI(4,5)P2/PLC pathway in B16-F10 and Mel 13 melanoma cell lines

**Fig. 6:**
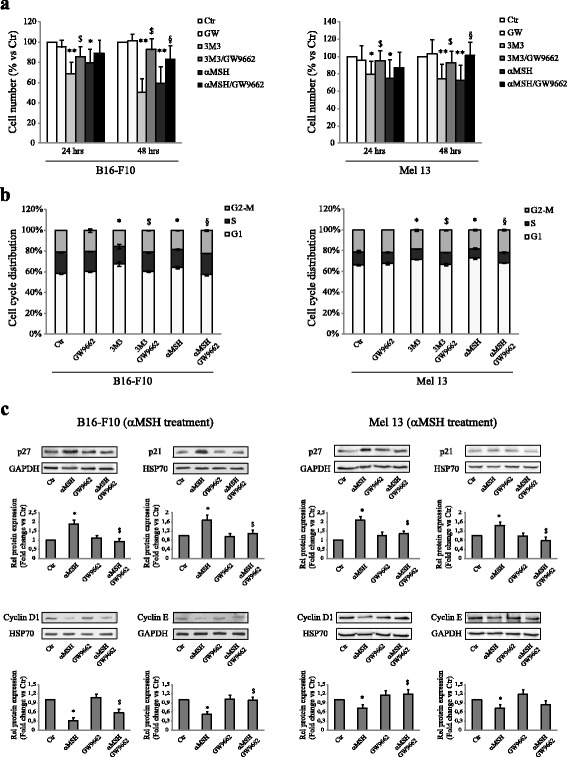
The PPARγ acts as an effector element in determining the reduction of cell number induced by the activation of PI(4,5)P2/PLC pathway in B16-F10 and Mel 13 melanoma cell lines. **a** Analysis of cell number performed on B16-F10 and Mel 13, after treatment for 24 and 48 h with 15 μM 3 m3 or 10^−7^ M αMSH in the presence or absence of 3 μM GW9662. In the combined treatment αMSH/GW9662 or 3 m3/GW9662, cells underwent a pre-treatment with GW9662 for 1 h. Cell number was expressed as a percent variation in comparison to the value of untreated control cells. Data are mean values ± SD of three independent experiments performed in triplicate. *p < 0.01 and **p < 0.001 (vs untreated cells); ^$^ *p* < 0.01 (vs 3 m3-treated cells); ^§^ *p* < 0.01 (vs αMSH-treated cells). **b** Cell-cycle distribution evaluated by flow cytometric analysis on B16-F10 and Mel 13, after treatment for 24 h with 15 μM 3 m3 or 10^−7^ M αMSH in the presence or absence of 3 μM GW9662. In the combined treatment αMSH/GW9662 or 3 M3/GW9662, cells underwent a pre-treatment with GW9662 for 1 h. The bar graph shows the distribution of cells among the different phases of the cell cycle. Data are mean values ± SD of three independent experiments performed in duplicate. *p < 0.01 (vs untreated cells); ^$^ *p* < 0.01 (vs 3 m3-treated cells); ^§^ *p* < 0.01 (vs αMSH-treated cells). **c** Western blot analysis of p27 (4 h), p21 (48 h), cyclin D1 and cyclin E (6 h) protein expression on cell lysate of B16-F10 and Mel 13, treated with 10^−7^ M αMSH in the presence or absence of 3 μM GW9662. In the combined treatment αMSH/GW9662, cells underwent a pre-treatment with GW9662 for 1 h. GAPDH or HSP70 were used as an equal loading control. Results refer to three independent experiments. Representative blots are shown. Densitometric scanning of band intensities was performed to quantify the change of protein expression (control value taken as one fold in each case). ^*^ *p* < 0,01 (vs untreated cells), ^$^ *p* < 0,01 (vs αMSH-treated cells)

In order to strengthen the role of PPARγ in mediating the reduction in the number of cells induced by the stimulation of “PI(4,5)P2/PLC pathway”, both B16-F10 cells and Mel 13 human melanoma cells were stimulated with 3 M3, for 24 and 48 h, in the presence or absence of GW9662, as an inhibitor of PPARγ [29]. This pattern of stimulation was compared to that represented by cells treated with αMSH, in the presence or absence of GW9662. In both cell lines, the exposure of cells to 3 M3 or αMSH led to a significant decrease of number of cells. Whereas, the same treatments, in the presence of GW9662, significantly hindered the effectiveness of 3 M3 or αMSH in reducing the cell number after 24 h of treatment (Fig. 6a). Consistently with these results, the exposure of B16-F10 and Mel 13 for 24 h to 3 M3 or αMSH, was able to promote a significant increase in the proportion of cells distributed in the G0/G1 phase of the cell cycle and the co-treatment with GW9962 counteracted the effects of both the agents (Fig. 6b). Accordingly, the inhibition of PPARγ by GW9662 reverted the effects on cell cycle modulators (p27, p21, Cyclin D1 and cyclin E) promoted by αMSH in both melanoma cell lines (Fig. 6c). All together, these results underline the key role of PPARγ in mediating the αMSH dependent effect on proliferation slow down.

## Discussion

MC1R is primarily located on the surface of melanocytes and plays an important role in the control of normal pigmentation. However, as a member of “G protein-coupled receptors” family, MC1R exerts a role in the control of proliferation. Studies on this topic are sporadic ad provide evidence that the activated receptor appears to be influenced by the physiological state and/or transformation. This study tried to confirm the different proliferative behavior, in response to MSH, in healthy and then in melanoma condition and to deepen the knowledge of the molecular basis responsible for the down-proliferative response of melanoma cells after exposure to αMSH.

The first series of experiments evidenced that while in primary cultures of human melanocytes the αMSH acted as a mitogenic agent [1, 3], in Mel 13 human melanoma cell line and in the B16-F10 murine melanoma cell line, this hormone did not exert the same effect. In the case of melanocyte cultures, the hyper-proliferation in response to αMSH stimulus can be found in literature [1, 3, 15–17]. This kind of response contravenes one of the axioms of cell biology, which associates the differentiation process with a decrease in proliferation rates. This proliferative reduction is usually considered necessary, in order to store energy, which can be employed to accomplish differentiation itself. Whereas, in the case of melanocytes, the hyper-proliferative response to the hormone may be interpreted as a necessity for melanocytes to increase their number, to produce more melanin and better protect the skin from ultraviolet radiation. On the other hand, melanoma cells have a basal sustained rate of proliferation, and our results highlight the ability of αMSH to trigger a mechanism capable of counteracting this biological feature. In fact, the slow down of proliferation that we previously described in B16-F10 [7], was confirmed on the Mel 13 human melanoma cell line. Thus, the effect promoted by αMSH on proliferation seems to be associated with the pathological state and not with the animal species which the culture derives from.

In order to find the pathway driving the αMSH mediated effects on proliferation in our cell lines, we treated cells with FSK, which is able to by-pass the MC1R receptor and to stimulate the overall cAMP/PKA cell potential [8]. It is noteworthy that in our cell lines FKS determined uniquely a hyper-proliferative response, both in NHMs and in melanoma cell lines. This result, which requires in depth study, may be ascribed to a CREB mediated mechanism. In fact, this transcription factor, one of the major PKA substrates, binds to the promoter of cell cycle regulators, such as cyclin D1, activating their transcription [32]. Furthermore, the comparison between the response of melanocytes and transformed cells to FSK showed a quantitative rather than a qualitative difference. The hyper-proliferative response to FSK appeared more marked in primary cultures of melanocytes than in the transformed condition. In fact, PKA is active in basal conditions and supported the rate of basal proliferation, in melanoma cells [33]. Therefore PKA activity cannot be further enhanced by additional stimulation, such as that due to FSK.

To define an alternative pathway which could justify the proliferation slow down induced by αMSH in B16-F10 and Mel 13 melanoma cell lines, our attention focused on the PI(4,5)P2/PLC pathway. This lipid involving pathway was in fact promoted in response to αMSH exposure in B16-F10 and was directly involved in the PPARγ activation [7]. When the PPARγ transcription factor is activated with lipid ligands or pharmacological activators, it promotes differentiation, anti-inflammatory effects and a slow down in the rate of cell proliferation. For this reasons it is considered a good candidate in the creation of possible innovative biological anticancer therapies [11, 12, 34–36]. First, we verified that the direct stimulation of PLC was able to promote PPARγ translocation into the nucleus as an active transcription factor, on B16-F10 and Mel 13 cell line. Then we found that the direct induction of the PI(4,5)P2/PLC pathway was able to reproduce the αMSH mediated effects on proliferation. Subsequent experiments, carried out by stimulating the cells with 3 M3 (or αMSH), after inhibition of PPARγ with GW9662, led to similar results in the two conditions, thereby highlighting the role of PPARγ as an effector element (triggered by the active PI(4,5)P2/PLC pathway) capable of mediating the phenomenon.

For what concerns the molecular nature of the proliferation slow down consequent to the activation of the αMSH/PPARγ connection, we showed that it is due to cell cycle withdrawal. Cell cycle progression is coordinated by the expression and activity of key regulators, such as cyclin dependent kinase (CDKs) and cyclins, including cyclin D, cyclin E [21, 22] and CDKs-inhibitors, like p21 and p27 [24, 25]. Our analyses showed that the G0/G1 arrest, mediated by the αMSH/PPARγ axis, is associated with an up-regulation of p21 and p27 and down-modulation of cyclin D1 and cyclin E. These results are in agreement with previous reports, employing natural and synthetic PPARγ agonists [34, 37–39]. p27 and p21 are transcriptional repressors of the same genes and associate with the same multi-protein complexes. It has recently been demonstrated that p27 and p21 collaborate in a sequential manner in gene repression during G1 phase [25]. The peaks of expression of p27 and p21, which we have observed at different times (4 h for p27 and 48 h for p21), could reflect their sequential function over time.

## Conclusion

This work dealt with the analysis of the αMSH/MC1R interaction for its ability to influence proliferation in healthy melanocytes and in melanoma cells, an aspect that has already been investigated in the past in preliminary and sporadic studies. This study attributes an opposite functional significance to the cAMP/PKA pathway and to the PLC/PPARγ axis, respectively, in the control of proliferation, giving the former a role in stimulating hyper-proliferation and the latter a function in promoting down-proliferation. This evidence attributes a dynamic role to the activated MC1R which is able to transducer effects on proliferation through these pathways. The αMSH dependent cAMP/PKA pathway activation favors hyper-proliferation and ensures the promotion of melanogenesis, in healthy melanocytes, which are poorly represented within the skin. In melanoma cells, in which the cAMP/PKA pathway is already active and correlates with their basal rate of proliferation, the PLC-PPARγ pathway emerged with particular emphasis, showing it exerts an opposite effect on proliferation. The PLC/PPARγ connection has both an anti-inflammatory and anti-cancer potential. In fact, acting as a transcription factor, PPARγ, is capable of enhancing both these activities. In summary, these results provide information on the biology of MC1R and the ability of this receptor to control extra-pigmentary functions. The PLC/PPARγ axis, inside the MC1R transduction machinery, could represent an element which potentially offers new therapeutic approaches for melanoma.

## Additional files

Additional file 1: Supplementary Materials and Methods. (DOCX 11 kb)

Additional file 2: Figure S1. Expression and functionality of MC1R in NHMs, B16-F10 and Mel 13 melanoma cell lines (**a**) Expression of *MC1R* mRNA evaluated by quantitative real-time RT-PCR. Values are normalized against the expression of *β-actin*. The values reported represent means ± SD of three independent experiments performed in triplicate. (**b**) Immunofluorescence analysis of MC1-R in NHM 1, NHM 2, B16-F10 and Mel 13. Nuclear staining with DAPI. Scale bar: 20 μM. (**c**) Western blot analysis of tyrosinase protein expression on cell lysate of NHM1 and NHM2 primary cultures of human melanocytes, B16-F10 and Mel 13, treated with 10^−7^ M αMSH for 72 h in NHM 1 and NHM 2, for 24 h in B16-F10 cells and for 48 h in Mel 13, respectively. GAPDH was used as an equal loading control. Results refer to three independent experiments. Representative blots are shown. Densitometric scanning of band intensities was performed to quantify the change of protein expression (control value taken as one fold in each case). *p < 0.01 (vs untreated cells). (PDF 9380 kb)

Additional file 3: Figure S2. Analysis of 3 M3 mediated calcium fluxes in (a) B16-F10 cells and (b) Mel 13 The profile of the intra-cytoplasm calcium fluxes in response to stimulation with 15 μM 3 M3 was obtained using a fluorimetric detection. The analysis was followed for 30 min by monitoring calcium fluxes each minute. The calcium fluxes promoted by 3 M3 were significantly higher (*p* < 0.01) than the baseline of untreated cells (100%). Results represent the mean ± SD of six experiments performed in exaplicate and are expressed as the percentage of fluo-3 fluorescence with respect to untreated cells (100%). (PDF 525 kb)

## Abbreviations

FSK: Forskolin MC1R, Melanocortin-1 Receptor
PPARγ: Peroxisome Proliferator Activated receptor-gamma
SDS-PAGE: Sodium dodecyl sulfate polyacrylamide gel electrophoresis
αMSH: alpha Melanocyte Stimulating Hormone
