# Supplementary material for: The α-melanocyte stimulating hormone/peroxisome proliferator activated receptor-γ pathway down-regulates proliferation in melanoma cell lines
Source: J Exp Clin Cancer Res. 2017 Oct 11;36:142. doi: 10.1186/s13046-017-0611-4 (PMC5637056; doi:10.1186/s13046-017-0611-4)
Supplement: Supplementary file 1 — Supplementary Materials and Methods. (DOCX 11 kb) [file 13046_2017_611_MOESM1_ESM.docx]

**Supplementary Materials and Methods**

**Genetic characterization of *MC1R***

The presence of polymorphic variants in the MC1R gene were excluded by DNA sequencing following PCR amplification using GGCAGCACCATGAACTAAGCAGG (forward) and GGACCAGGGAGGTAAGGAAC (reverse) primers of genomic DNA, both in human melanoma cells and primary cultures of human melanocytes, obtained from cell cultures with DNeasy Blood and Tissue kit from Qiagen (Qiagen, Milan, Italy). DNA fragments were sequenced in both strands by automated sequencing.

**Calcium measurement**

The calcium measurement was performed according to a protocol supplied by Beckman Coulter (Milan, Italy; Application –Information A-2049A by Jacab M, Ritter M, Moritz S and Hundsberger H). Cells were plated in a 24-well plate at a density of 8 X 10^4^ cells/well and grown overnight. Cells were loaded with the calcium-sensitive dye fluo-3 AM (Sigma-Aldrich Srl, Milan, Italy) at a concentration of 1.5 μM (in loading buffer, containing 135 mM NaCL, 4.5mM KCl, 1.5 mM CaCl2, 0.5 mM MgCl2, 10 mM HEPES, 5.6 mM glucose, pH 7.4) for 1 h at room temperature in the dark. Cells were washed once with a loading buffer and then treated with the different stimuli, in accordance with the experimental design. Immediately after the treatment, calcium was measured with a DTX 880 multimode-detector controlled by multimode detector analysis software version 2.0 (Beckman Coulter) (Beckman Coulter, Milan, Italy). Experiments were performed in the fluorescence intensity bottom reading mode, with the appropriate filter setting according to the absorption and maximum emission of fluo-3AM (506 and 526 mm, respectively). Kinetic scans (30 cycles, 60-s intervals) were performed to assess the time-course of calcium change. Results are the mean of six independent experiments in exaplicate and were expressed as % of fluo-3 fluorescence with respect to the baseline of untreated cells.
