# Supplementary figures and images for: The α-melanocyte stimulating hormone/peroxisome proliferator activated receptor-γ pathway down-regulates proliferation in melanoma cell lines
Source: J Exp Clin Cancer Res. 2017 Oct 11;36:142. doi: 10.1186/s13046-017-0611-4 (PMC5637056; doi:10.1186/s13046-017-0611-4)

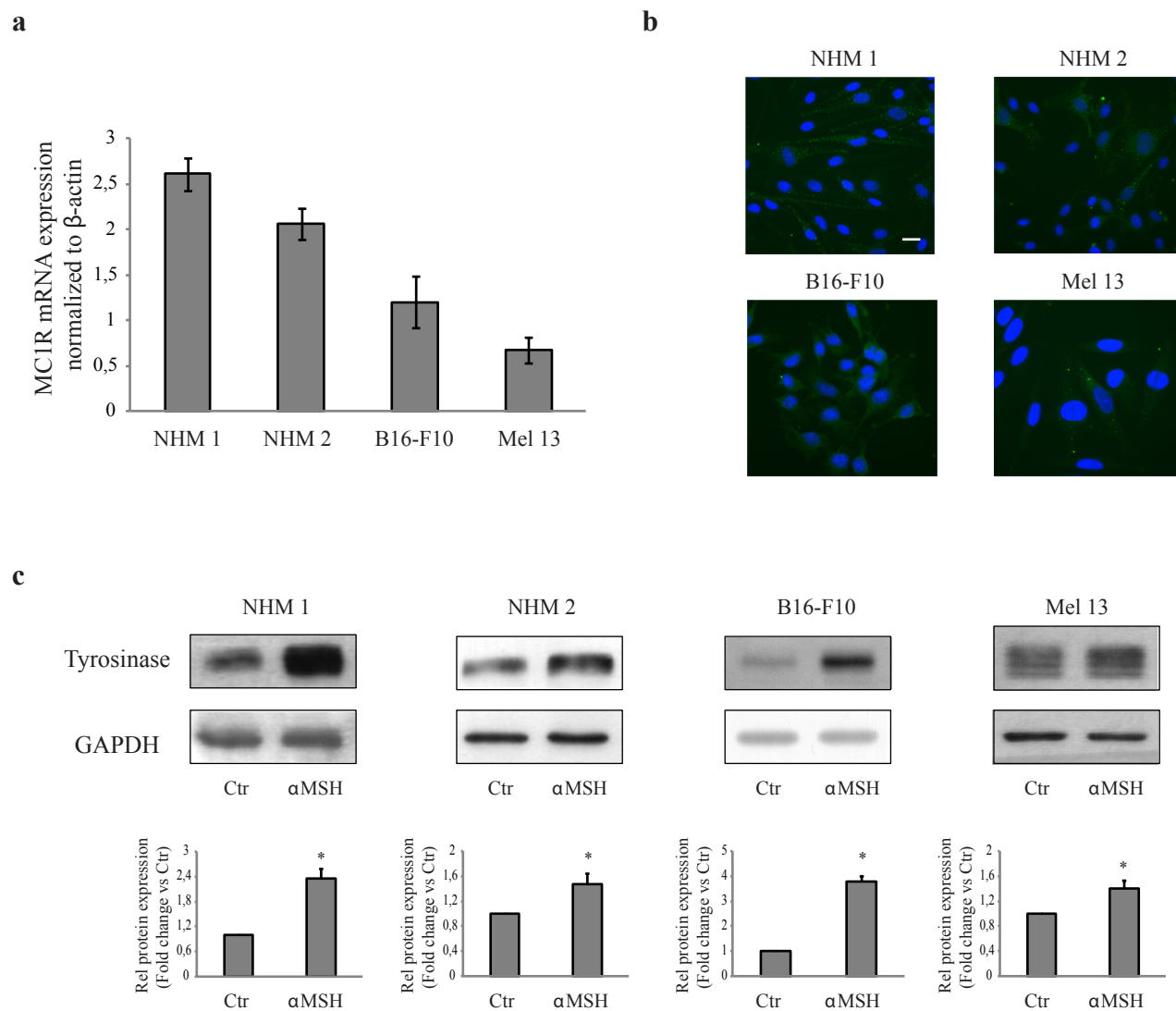

Figure S1

Supplement: Supplementary file 2 — Expression and functionality of MC1R in NHMs, B16-F10 and Mel 13 melanoma cell lines (a) Expression of MC1R mRNA evaluated by quantitative real-time RT-PCR. Values are normalized against the expression of β-actin. The values reported represent means ± SD of three independent experiments performed in triplicate. (b) Immunofluorescence analysis of MC1-R in NHM 1, NHM 2, B16-F10 and Mel 13. Nuclear staining with DAPI. Scale bar: 20 μM. (c) Western blot analysis of tyrosinase protein expression on cell lysate of NHM1 and NHM2 primary cultures of human melanocytes, B16-F10 and Mel 13, treated with 10−7 M αMSH for 72 h in NHM 1 and NHM 2, for 24 h in B16-F10 cells and for 48 h in Mel 13, respectively. GAPDH was used as an equal loading control. Results refer to three independent experiments. Representative blots are shown. Densitometric scanning of band intensities was performed to quantify the change of protein expression (control value taken as one fold in each case). *p < 0.01 (vs untreated cells). (PDF 9380 kb) [file 13046_2017_611_MOESM2_ESM.pdf]

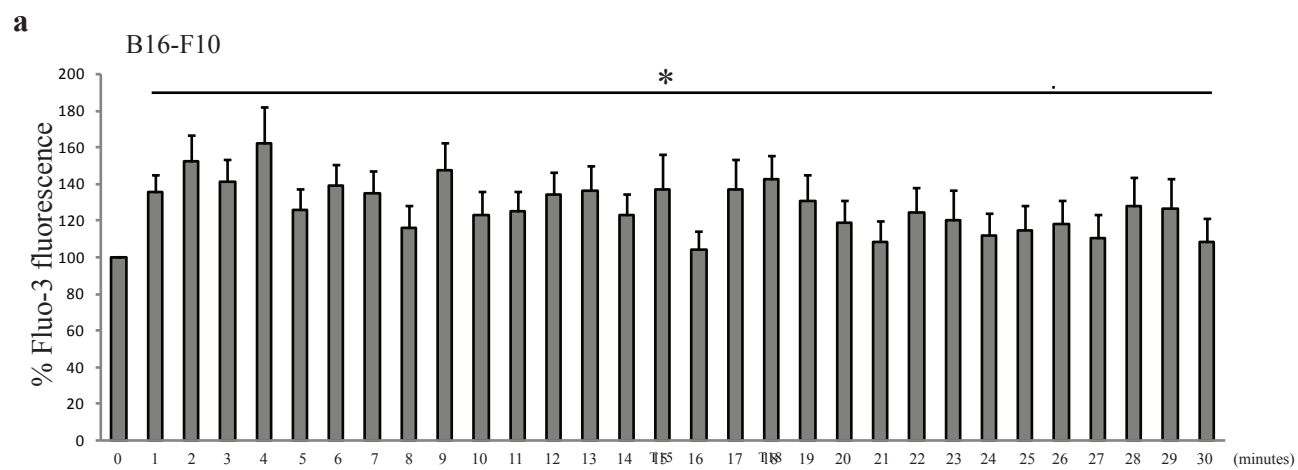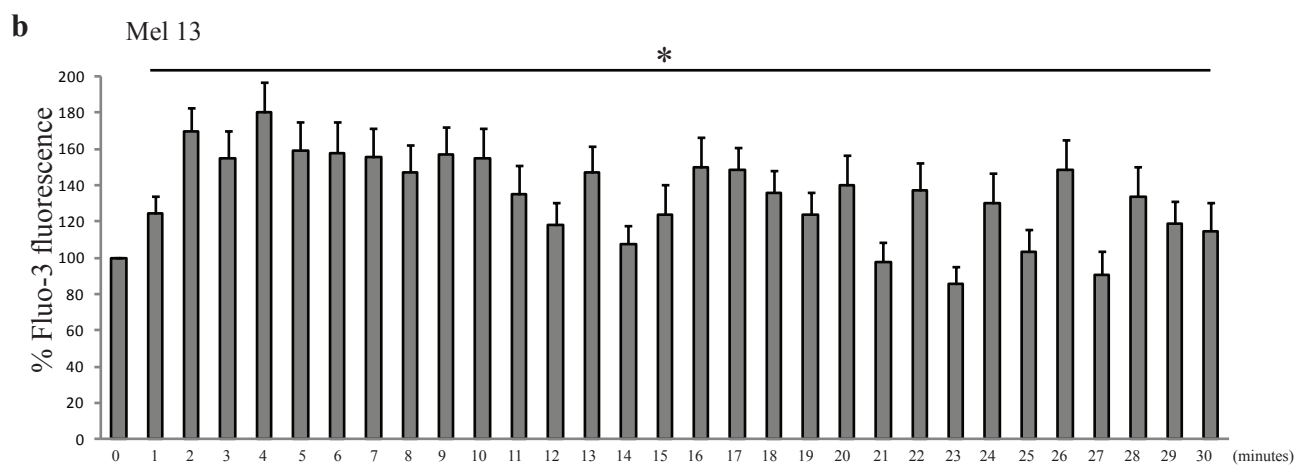

Figure S2

Supplement: Supplementary file 3 — Analysis of 3 M3 mediated calcium fluxes in (a) B16-F10 cells and (b) Mel 13 The profile of the intra-cytoplasm calcium fluxes in response to stimulation with 15 μM 3 M3 was obtained using a fluorimetric detection. The analysis was followed for 30 min by monitoring calcium fluxes each minute. The calcium fluxes promoted by 3 M3 were significantly higher (p < 0.01) than the baseline of untreated cells (100%). Results represent the mean ± SD of six experiments performed in exaplicate and are expressed as the percentage of fluo-3 fluorescence with respect to untreated cells (100%). (PDF 525 kb) [file 13046_2017_611_MOESM3_ESM.pdf]
